# Breast Cancer and African Ancestry: Lessons Learned at the 10-Year Anniversary of the Ghana-Michigan Research Partnership and International Breast Registry

**DOI:** 10.1200/JGO.2015.002881

**Published:** 2016-07-27

**Authors:** Evelyn Jiagge, Joseph Kwaku Oppong, Jessica Bensenhaver, Francis Aitpillah, Kofi Gyan, Ishmael Kyei, Ernest Osei-Bonsu, Ernest Adjei, Michael Ohene-Yeboah, Kathy Toy, Karen Eubanks Jackson, Marian Akpaloo, Dorcas Acheampong, Beatrice Antwi, Faustina Obeng Agyeman, Zainab Alhassan, Linda Ahenkorah Fondjo, Osei Owusu-Afriyie, Robert Newman Brewer, Amma Gyamfuah, Barbara Salem, Timothy Johnson, Max Wicha, Sofia Merajver, Celina Kleer, Judy Pang, Emmanuel Amankwaa-Frempong, Azadeh Stark, Francis Abantanga, Lisa Newman, Baffour Awuah

**Affiliations:** **Evelyn Jiagge**, **Joseph Kwaku Oppong**, **Francis Aitpillah**, **Ishmael Kyei**, **Ernest Osei-Bonsu**, **Ernest Adjei**, **Marian Akpaloo**, **Dorcas Acheampong**, **Beatrice Antwi**, **Faustina Obeng Agyeman**, **Zainab Alhassan**, **Osei Owusu-Afriyie**, **Amma Gyamfuah**, **Emmanuel Amankwaa-Frempong**, **Francis Abantanga**, and **Baffour Awuah**, Komfo Anokye Teaching Hospital; **Linda Ahenkorah Fondjo**, Kwame Nkrumah University of Science and Technology, Kumasi; **Michael Ohene-Yeboah**, University of Ghana Medical School and Korle-Bu, Accra, Ghana; **Evelyn Jiagge**, **Jessica Bensenhaver**, **Kathy Toy**, **Robert Newman Brewer**, **Timothy Johnson**, **Max Wicha**, **Sofia Merajver**, **Celina Kleer**, **Judy Pang**, and **Lisa Newman**, University of Michigan Medical School, Ann Arbor; **Jessica Bensenhaver**, **Kofi Gyan**, **Barbara Salem**, **Azadeh Stark**, and **Lisa Newman**, Henry Ford Health System International Center for the Study of Breast Cancer Subtypes, Detroit, MI; and **Karen Eubanks Jackson**, Sisters Network, Houston, TX.

## Abstract

Women with African ancestry in western, sub-Saharan Africa and in the United States represent a population subset facing an increased risk of being diagnosed with biologically aggressive phenotypes of breast cancer that are negative for the estrogen receptor, the progesterone receptor, and the *HER2*/*neu* marker. These tumors are commonly referred to as triple-negative breast cancer. Disparities in breast cancer incidence and outcome related to racial or ethnic identity motivated the establishment of the International Breast Registry, on the basis of partnerships between the Komfo Anokye Teaching Hospital in Kumasi, Ghana, the University of Michigan Comprehensive Cancer Center in Ann Arbor, Michigan, and the Henry Ford Health System in Detroit, Michigan. This research collaborative has featured educational training programs as well as scientific investigations related to the comparative biology of breast cancer in Ghanaian African, African American, and white/European American patients. Currently, the International Breast Registry has expanded to include African American patients throughout the United States by partnering with the Sisters Network (a national African American breast cancer survivors’ organization) and additional sites in Ghana (representing West Africa) as well as Ethiopia (representing East Africa). Its activities are now coordinated through the Henry Ford Health System International Center for the Study of Breast Cancer Subtypes. Herein, we review the history and results of this international program at its 10-year anniversary.

## INTRODUCTION

**Fig 1:**
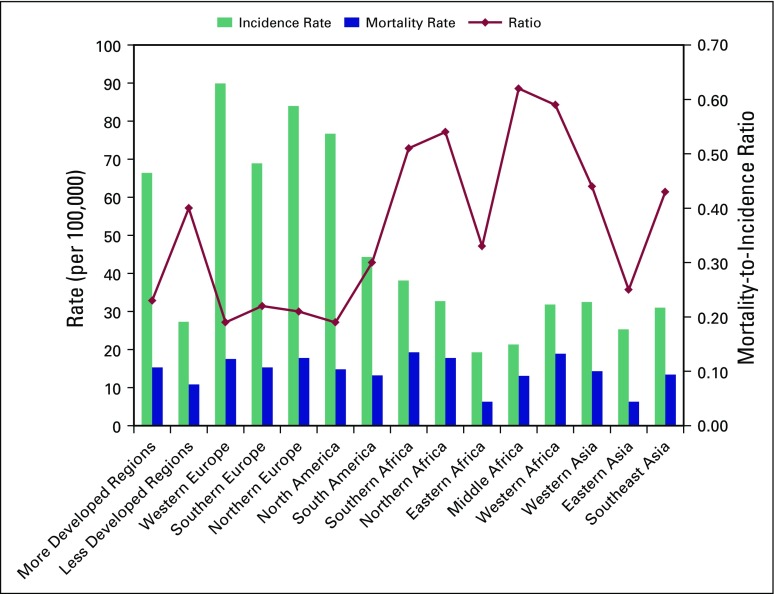
International variation in breast cancer incidence, mortality, and mortality-to-incidence ratios. Data from Jemal et al,^7^ Newman et al,^9^ and Jiagge et al.^10^

Financial constraints are a constant reality in low- and middle-income countries and pose enormous barriers to both quantifying and addressing the cancer burden in sub-Saharan Africa. The limited data available have been generated by the Globocan 2008 database of the International Agency for Research on Cancer and from recent attempts to report population-based cancer incidence rates from selected countries such as Uganda and Ghana.^1-6^ Taken together, these resources suggest that breast cancer is an increasing problem. Likely explanations for breast cancer as an increasing health threat include more prolonged longevity in many African communities (because breast cancer risk increases as women age); acceptance of Westernized, higher fat dietary patterns (which can increase breast cancer risk in both pre- and postmenopausal women); and increased adoption of reproductive patterns that are more prevalent in Western populations (such as delayed childbearing and reduced overall parity, which are risk factors that increase incidence of hormone receptor–positive breast cancer). Increasing rates of breast cancer cases are particularly alarming within most sub-Saharan African countries, where the already overburdened health care system is unequipped to afford early detection and multidisciplinary treatment programs. The disturbing and excessively high mortality-to-incidence ratios of breast cancer in sub-Saharan Africa compared with other parts of the world are depicted in Figure 1.

In the United States, race- and/or ethnicity-associated disparities in breast cancer incidence and outcome have been documented for many decades. Breast cancer mortality rates are disproportionately higher for African American compared with white/European American women, and African American women tend to be diagnosed with breast cancer at younger ages. Furthermore, although the population-based breast cancer incidence rates are increasing among African American women, the survival disparity is widening, with current data revealing a 42% higher mortality rate among African Americans.^11^ The mortality differences have historically been ascribed to socioeconomic differences, because poverty rates and barriers to accessing adequate health care are more prevalent among African Americans. However, the younger age distribution for breast cancer, studies documenting higher mortality risk after adjusting for socioeconomic status, and the two-fold higher population-based incidence rates of the biologically aggressive triple-negative breast cancer (TNBC; tumors that are negative for estrogen receptor, progesterone receptor, and *HER2*/*neu*) phenotype within the African American community have fueled speculation that African ancestry itself might be associated with hereditary susceptibility for specific patterns of breast cancer.^12-14^

These issues provided the rationale for establishing the International Breast Registry (IBR), involving a breast cancer research partnership between the Komfo Anokye Teaching Hospital (KATH), the University of Michigan (UM), and the Henry Ford Health System (HFHS). Most recently, this program has evolved into the HFHS International Center for the Study of Breast Cancer Subtypes. Exploratory conversations and bicontinental introductory visits occurred in 2004 to 2005, and the institutional review boards of each institution provided their initial human research ethics approvals in 2006. The early goals of this collaborative were therefore related to studying the biology of breast cancer in women with African ancestry, and indeed, the first joint publication from this team was an article providing how-to guidelines for other investigators regarding the conduct of cancer research in developing, low- and middle-income countries.^15^

The IBR has grown enormously since its inception. Although this collaborative continues to feature a robust breast tumor repository that has provided provocative, hypothesis-generating data regarding breast cancer in Ghanaian African as well as African American women, it has also expanded its portfolio of educational and training exchange programs. In this review, we summarize the various outcomes of this international effort over the past 10 years, as presented in peer-reviewed publications and academic meeting abstracts. We also review the non–research-related productivity of this partnership, featuring investment in the oncology services infrastructure of Ghana.

## BREAST CANCER AND AFRICAN ANCESTRY: PATTERNS IDENTIFIED THROUGH THE IBR

The concept of subtyping breast cancer has assumed increasing importance as our knowledge of targeted therapy has advanced. Invasive breast cancers that are positive for the estrogen receptor and/or the progesterone receptor can be managed systemically with a variety of endocrine agents, such as tamoxifen for premenopausal patients and tamoxifen or one of the aromatase inhibitors for postmenopausal patients. Tumors that overexpress HER2/*neu* benefit greatly from targeted, anti-HER2 therapy such as trastuzumab and/or pertuzumab. Equally important and relevant to treatment planning is the fact that these targeted agents are contraindicated in patients whose tumors are negative for these markers, and using them in patients with TNBC results in exposing patients unnecessarily to the toxicity of an ineffective regimen. Studies of breast cancer in the United States have revealed that frequency and population-based incidence rates of TNBC are significantly higher in African American women compared with women of other racial or ethnic identities.^16,17^ The IBR has investigated the hypothesis of inherited TNBC susceptibility associated with African ancestry by comparing breast tumor phenotypes in African American and western, sub-Saharan African women, because these two population subsets have shared ancestry resulting from the colonial-era slave trade.^14^

Therefore, one of the initial publications of the KATH-UM IBR collaborative focused on comparisons of TNBC prevalence in white American women compared with African American and Ghanaian women. The HFHS, which provides care to the robustly diverse metropolitan Detroit, MI, community, served as the source for the comparison patient population. This study revealed that TNBC accounted for the majority of KATH patients with breast cancer.^18^ Subsequent studies from the IBR tissue repository, on the basis of larger sample sizes, have confirmed these results. The most recent analyses, on the basis of immunohistochemistry studies of 234 Ghanaian patients with breast cancer (with complete marker profiling on 173 invasive tumors), revealed that 92 (53.2%) of 173 tumors were triple negative.^19^ In contrast, the lowest frequency of TNBC was seen in white American patients (15.5%), and TNBC frequency for African American patients was intermediate between these two extremes at 30%. This pattern persisted in subset analyses of patients younger than age 50 years. These findings are consistent with the theory that extent of African ancestry correlates with likelihood of being diagnosed with TNBC, because African American patients represent a genetically admixed population. Table 1 lists the results of studies characterizing the breast cancer burden of Ghanaian women on the basis of data from the IBR research collaborative.

**Table 1:**
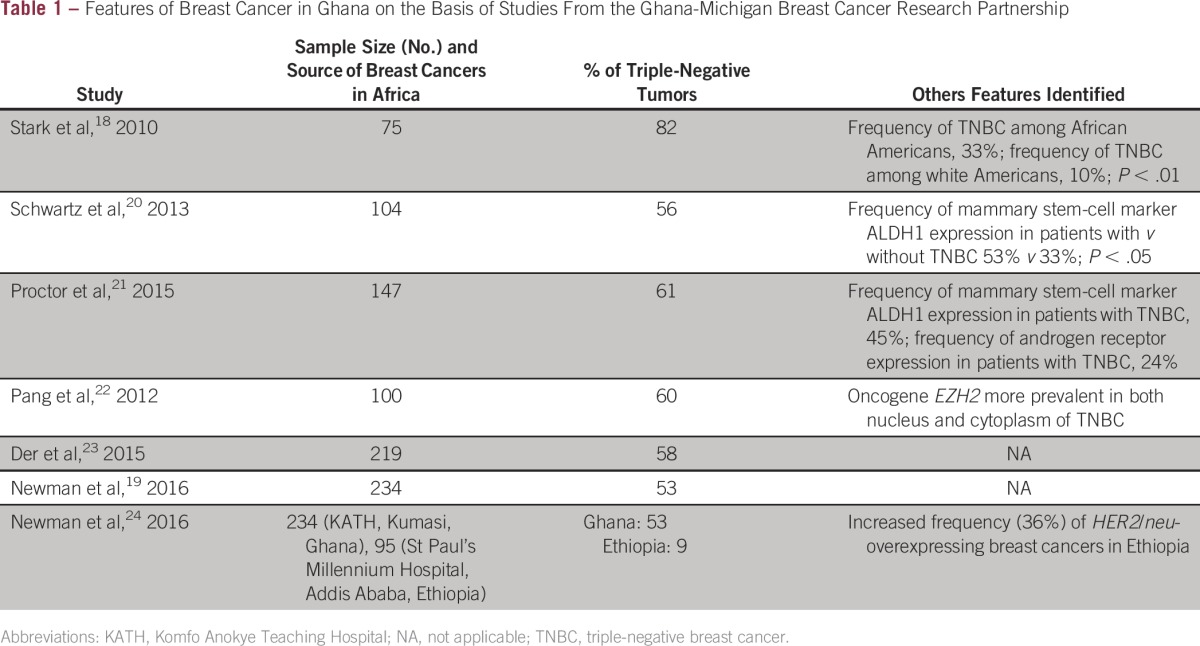
Features of Breast Cancer in Ghana on the Basis of Studies From the Ghana-Michigan Breast Cancer Research Partnership

Although studies from the IBR have been enlightening with regard to understanding the breast cancer burden of western sub-Saharan Africa as exemplified by Ghanaian women, expansion of the registry has provided opportunities to study breast cancer in East Africa as well. Contributions from partners at the St Paul’s Millennium Hospital in Addis Ababa, Ethiopia, have yielded interesting preliminary findings regarding breast tumor phenotypes from Ethiopian women. Immunohistochemistry performed at UM on 95 invasive breast cancers from Addis Ababa revealed a low frequency of TNBC (9%).^24^ The history of population migrations through the African diaspora and shared ancestry may explain these disparate results regarding the prevalence of TNBC in African American and Ghanaian women compared with Ethiopians. The colonial-era slave trade that was controlled by Europeans for the purpose of generating a labor supply in North America focused on capture and enforced trans-Atlantic transport of West Africans.^25^ In contrast, enslavement of East Africans from the region of Ethiopia often involved Arabic slave traders and enforced migration to northern Africa and Asia.^26,27^ Therefore, African American women are more likely to have shared ancestry with women from western Africa, and this has been confirmed by genotyping studies of markers associated with geographic ancestry, commonly referred to as ancestry informative markers.^28-30^

Studies of breast tumor biology have generated the stem-cell theory, which hypothesizes that mammary tumor virulence and metastatic risk are driven by a small subset of cells within the cancer known as the stem cells. Thus, efforts to identify and characterize the mammary stem cells represent an exciting body of research. UM researchers have pioneered studies of the mammary stem-cell hypothesis and have reported on aldehyde dehydrogenase-1 (ALDH1) expression as a reliable, immunohistochemically detectable marker of the mammary stem cell and have also reported its association with more virulent tumors.^31^ Studies of KATH tumors at UM demonstrated elevated expression of this marker in both benign and malignant breast tissue from Ghanaian women.^20^

The polycomb group protein EZH2 is another molecule that has been implicated in mammary stem cells and TNBC progression, as demonstrated by UM researchers involved with the IBR.^32,33^ Therefore, we incorporated studies of EZH2 into the IBR. This marker was found to have unique patterns of expression in Ghanaian breast specimens studied through the KATH-UM partnership.^22^

Lastly, TNBC subtyping is a promising avenue for gaining insights regarding more refined, personalized treatment of TNBC.^34-37^ Existing research suggests that there are at least six different TNBC subtypes, and some of the distinguishing features are related to luminal-like characteristics seen in the androgen receptor subtype versus the stem cell–like characteristics seen in the mesenchymal subtypes. Despite the disproportionately high frequency of TNBC among women with African ancestry, the TNBC subtyping research has been based almost exclusively on data sets representing white American, European, and Asian populations. Thus far, no data sets of gene expression profiles representing African patient populations have been available for inclusion in this body of research. These issues motivated exploratory analyses of tissue from the IBR looking at expression of both androgen receptor and ALDH1 as immunohistochemistry surrogates for TNBC subtypes. Among the Ghanaian tumors, an intriguing finding appeared suggesting a novel TNBC subtype, featuring coexpression of both androgen receptor and ALDH1.^38^

As noted earlier, the partnerships within the IBR oncology teams have generated valuable insights regarding breast cancer phenotypes that are more prevalent among women with West African ancestry, such as triple-negative tumors. Patient-derived xenografts (PDXs) represent an exciting research strategy for studying breast tumor biology and novel therapies. Implantation of breast cancer fragments into mice mammary fat pads yields a renewable supply of human tumors that can be used for a variety of in vivo rodent model experiments. Through the KATH-UM research partnership, a series of PDXs have been created on the basis of the tumors from Ghanaian, African American, and white American patients with breast cancer.

## BREAST CANCER IN GHANA: DIAGNOSTIC AND TREATMENT ADVANCES PROMOTED BY THE GHANA-MICHIGAN PARTNERSHIP

The IBR collaborative has also served as an investment in improving the clinical services available to Ghanaian women with breast problems. Historically, the KATH breast clinics relied predominantly on open surgical diagnostic biopsies to confirm or rule out the presence of cancer in any woman presenting with a breast abnormality or mass. The scheduling and implementation of a surgical diagnostic procedure are time consuming and use valuable, costly operating room resources. Furthermore, this sequence increases the risk that the affected patient (who has likely already traveled a distance and expended personal finances to seek medical attention) may be lost to follow-up. The early years of this partnership featured a training program in using percutaneous core needle biopsies to diagnose breast cancer on-site during the outpatient clinic visit. The success of this program in terms of accurately and efficiently establishing a diagnosis of breast cancer has been reviewed and documented.^39^

Multidisciplinary or multimodality treatment of breast cancer requires immunohistochemistry resources that can efficiently and accurately assess for expression of the estrogen receptor, the progesterone receptor, and HER2/*neu*. These markers are critical in being able to appropriately determine a patient’s response to endocrine therapy and/or targeted anti-HER2/*neu* therapy. Lacking this molecular marker information, patients with hormone receptor–negative breast cancer may face the toxicity of ineffective endocrine therapy, and patients with hormone receptor–positive breast cancer may miss the opportunity to receive life-saving endocrine therapy. Similarly, HER2/*neu* expression can identify tumors that are quite sensitive to chemotherapy and anti-HER2/*neu* agents. The Ghana-Michigan partnership featured a training program in immunohistochemistry for the KATH pathology team, as well as the development of a resource supply and allocation system that has enabled the KATH Breast Oncology Program to routinely generate their own molecular marker reports for each patient diagnosed with invasive breast cancer.

This partnership has also generated opportunities to use telemedicine technology for real-time international collaboration and multidisciplinary discussion. The UM partners invested in dedicated Internet and teleconference equipment on-site at KATH. This teleconference unit allows the Michigan-based and KATH teams to discuss patient care on a weekly basis and to share conference proceedings through live interactions.

Our Ghanaian partners have also been able to promote general breast health awareness programs as a consequence of the expanded breast cancer attention generated by this research collaborative. KATH principal investigator Baffour Awuah has worked with regional herbalists, primary care physicians, and nurses to conduct educational seminars promoting breast cancer early detection strategies. Lacking adequate financial resources as well as accurate population-based statistics on breast cancer incidence, it is not feasible to conduct community-wide mammographic screening programs. Therefore, these early detection programs largely focus on dissemination of information regarding clinical signs and symptoms of breast cancer (eg, dominant lump, bloody nipple discharge) and the importance of prompt biopsy with initiation of treatment. Although we cannot quantify the effectiveness of our efforts with regard to breast cancer stage distribution in Ghana, our KATH colleagues have established a tumor registry office (B. Awuah, personal communication, December 2014) and have seen increasing volumes of breast cancer annually as well as anecdotal observations of more women presenting with operable, earlier-stage disease. Strengthening this tumor registry (which has struggled with maintaining consistent personnel and completeness of data collection) remains a high priority for the Michigan-Ghana Collaborative, and we have also invested in advanced training of tumor registry personnel. Although lumpectomy and breast radiation are options that are available to patients with breast cancer patients in Ghana, few women present with tumors that are amenable to the breast conservation approach despite anecdotal observations of an earlier-stage distribution for KATH patients. Furthermore, concerns regarding inadequate pre- and postoperative mammographic imaging availability have generated suspicion that breast-conserving surgery cannot be planned with optimal information regarding extent of disease and adequacy of resection.

## BREAST CANCER AND AFRICAN ANCESTRY: THE GHANA-MICHIGAN PARTNERSHIP AS A MODEL FOR EXPANSION

The multifaceted success of the Michigan partnership with KATH has established the foundation for expansion and collaboration with other health care facilities in Africa. As a consequence, this program has grown, with exchange programs that have included St Paul’s Millennium Hospital in Ethiopia as well as the following three additional sites in Ghana: the Korle Bu Teaching Hospital in Accra, the Tamale Teaching Hospital in Tamale, and the Sunyani Teaching Hospital in Sunyani. Table 2 lists the studies that have been published and presented through these partnerships. International expansion efforts beyond Africa are also in development, as partnerships are being pursued with the All India Institute of Medical Sciences in New Delhi, India, and the Third Xiangya Hospital, Central South University in Hunan, China.

**Table 2:**
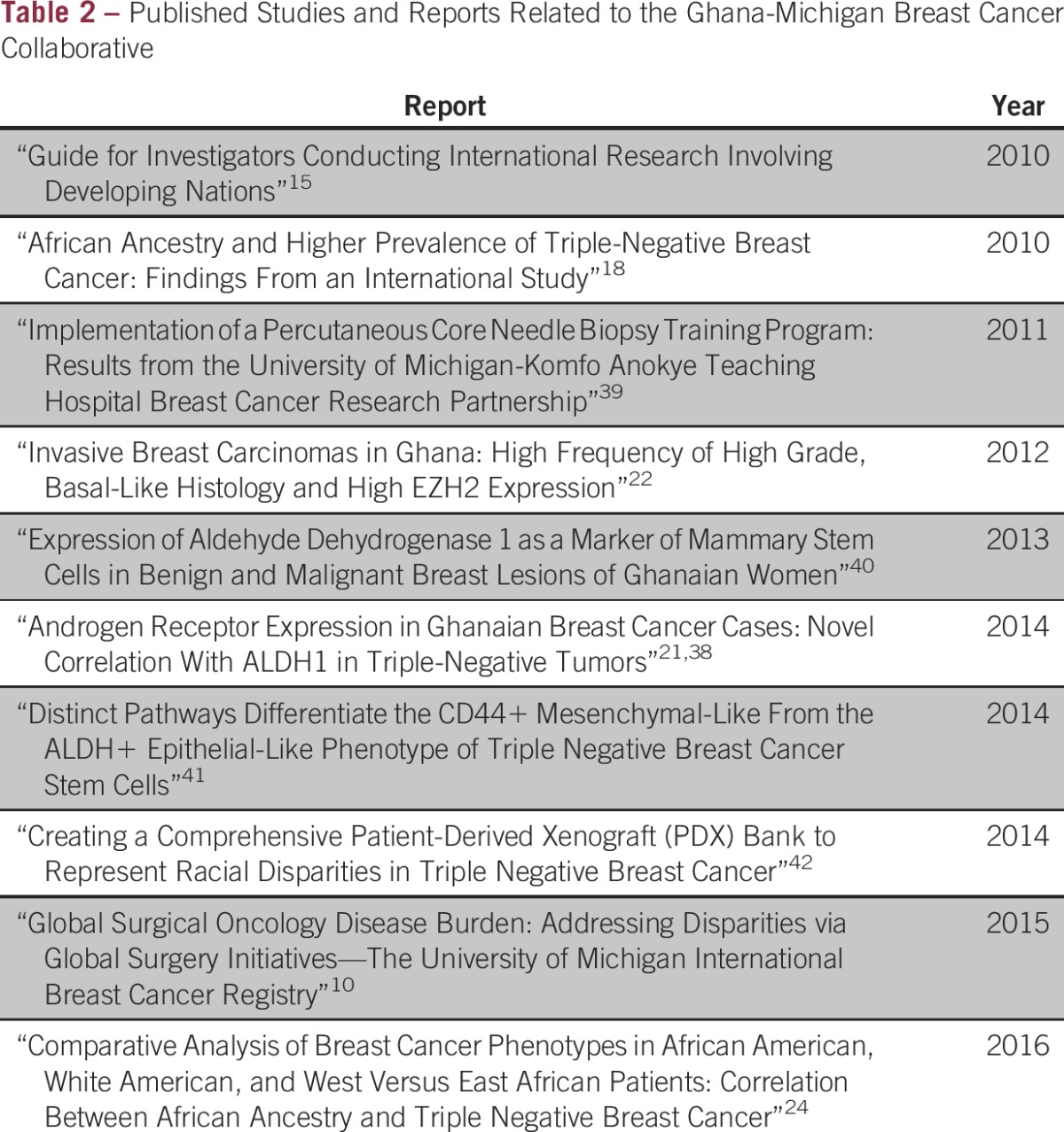
Published Studies and Reports Related to the Ghana-Michigan Breast Cancer Collaborative

The KATH-UM relationship has served as a platform for training the physician-scientists from Ghana. More than a dozen Ghanaians have spent time at UM for observorships and research programs. E.J. is a surgeon from KATH, currently completing her PhD work at UM in cancer biology, after which she will return to Ghana to assume leadership of a Ghana-based translational research program.

Within the United States, expansion of this registry has involved partnership with the Sisters Network (Houston, TX). This is a national organization of African American breast cancer survivors^43^ that currently has a membership of approximately 3,000 women in more than 30 chapters across 22 states. The Sisters Network membership contributes to the IBR by providing recruitment opportunities at their national as well as local meetings. Recruitment involves participants agreeing to provide access to medical records and saliva specimens suitable for DNA extraction and genotyping studies. Long-term goals of this registry include studies of germline breast cancer risk in women with diverse racial and ethnic backgrounds in the United States compared with international populations.

We enthusiastically look forward to this expanded research as a valuable contribution to precision medicine initiatives. We are also committed to ongoing investment in the breast oncology services available to patients in all of our partnering sites as well as throughout other low- and middle-income countries. Core biopsy programs, immunohistochemistry programs, and multidisciplinary tumor board conferences are examples of the services that are promoted through our research collaborative.

Other investigators with an interest in this type of international cancer research should be mindful of evolving policies that regulate transport of human tissue via commercial airlines. Updates on regulatory requirements and restrictions can be obtained at the Centers for Disease Control and Prevention Web site.^44^ Our group typically transported breast tissues for immunohistochemistry studies as formalin-fixed specimens embedded in paraffin blocks. The fresh specimens for PDX work were transported in dry ice. Commercial carriers have regulations for labeling and packaging of dry ice, including maximum limits for quantity of dry ice that can be transported. Updates on these regulations can be accessed through the particular carrier’s dangerous goods office and Web site. Investigators should also work with their institution’s liaison to the Occupational Safety and Health Administration for training in handling dry ice, and further information can be obtained from the Occupational Safety and Health Administration directly.

International partnerships represent a powerful and unique opportunity to advance insights regarding the etiology of domestic disparities in breast cancer burden related to racial and ethnic identity. These efforts also provide valuable cultural, academic, and educational exchange programs as well as opportunities to strengthen the oncology services in under-resourced countries. Our program is certainly not unique; medical missionary–type work has existed for many decades. Hopefully this summary of our 10-year experience with international breast cancer outreach and research will provide motivation for others to add to this growing field.
